# Reproductive desire among women living with HIV/AIDS in Central Brazil: Prevalence and associated factors

**DOI:** 10.1371/journal.pone.0186267

**Published:** 2017-10-20

**Authors:** Marise Ramos de Souza, Waldemar Naves do Amaral, Rafael Alves Guimarães, Giovanni Rezza, Sandra Maria Brunini

**Affiliations:** 1 Nursing Course, University Federal of Goiás, Jataí, Goiás, Brazil; 2 Medicine School, University Federal of Goiás, Goiânia, Goiás, Brazil; 3 Tropical Diseases and Public Health, University Federal of Goiás, Goiânia, Goiás, Brazil; 4 Department Infectious diseases, Istituto Superiore di Sanità, Rome, Italy; 5 Nursing School, University Federal of Goiás, Goiânia, Goiás, Brazil; National Institute of Health, ITALY

## Abstract

**Background:**

The purpose of this study was to estimate the prevalence and examine the factors associated with reproductive desire among women living with HIV/AIDS (WLWHA) in Central Brazil.

**Methods:**

A cross-sectional study involving 274 WLWHA, aged 18 to 49 years, was conducted with the support of treatment services and non-governmental organizations that assist people living with HIV/AIDS. Data regarding sociodemographic characteristics, substance use, sexual behavior, and reproductive variables were collected through interviews. Poisson regression with robust variance was used to analyze the factors associated with reproductive desire.

**Results:**

The prevalence of reproductive desire was 25.9% (95.0% confidence interval [CI]: 21.1–31.4%). This outcome was associated with age < 30 years (adjusted prevalence ratio [APR]: 2.93; p < 0.001), black skin color or race (APR: 2.28; p = 0.017), partner's reproductive desire (APR: 7.55; p < 0.001), absence of children (APR: 2.13; p = 0.003), history of abortion (APR: 1.65; p = 0.045) and undetectable viral load at the time of data collection (APR: 1.92; p = 0.043).

**Conclusion:**

The prevalence of reproductive desire among WLWHA was relatively high. It is necessary to include fertility issues as part of assistance and counseling for women in referral services to support them with their feelings, goals, and needs regarding reproductive choices.

## Introduction

Human immunodeficiency virus (HIV) infections continue to present alarming numbers worldwide, despite advances in preventive measures and antiretroviral therapy (ART). It is estimated that there are 36.7 million people living with HIV/AIDS (PLWHA) worldwide (17.8 million women). It is also estimated that 2.1 million new HIV infections occurred in 2015 at the global level [1].

It was estimated that in Brazil—a large country in South America—there were 830,000 PLWHA and 44,000 new infections in 2015 [2]. HIV prevalence in Brazil is estimated to be between 0.4–0.7% among individuals aged 15–49 years [3]. In 2015, 32,321 new cases of HIV infection were reported in Brazil. The distribution of these cases was 22,672 men and 9,639 women (sex ratio of 2.4:1). Of the total cases, 2,574 (8.0%) and 1,189 were reported in Midwest Brazil and the Goiás State, respectively [4].

Since the discovery of the virus, women living with HIV/AIDS (WLWHA) have been stigmatized and their desire to become mothers repressed. Concerns about the risk of vertical and horizontal transmission often overshadows the reproductive wishes of WLWHA [5]. However, since 1996, Brazil has been adopting important measures for the control and prevention of HIV such as free and universal access to ART, implementation of standard treatment protocols and actions to reduce the risk of mother-to-child transmission [6].

The profile of HIV has changed such that it has ceased to be incurable and has instead become a chronic illness with various treatment options; thereby preventing frequent opportunistic infections in PLWHA [7]. Couples living with HIV maintain an active sexual and reproductive life. They often wish to start a family and have children [8] and view conception as an important component of their social status and their life together as a couple [8–9]. Furthermore, the fear of motherhood among WLWHA has diminished owing to the effectiveness of ART, which has increased the life expectancy and improved the physical and mental well-being of these women. This has enabled WLWHA to led a lifestyle similar to that of uninfected people [8, 10, 11, 12, 13] with longer and healthier lives, enabling them to be more optimistic about their lives and fostered the desire to have children [14,15].

Studies have shown wide variations in the reproductive desire of WLWHA worldwide. In Canada, studies have shown variation in the frequency of reproductive desire from 25.8% to 69.0% and associations with variables such as age, ethnicity, regular partnership, and number of children were observed [16, 17]. In Spain, Hernando et al. [18] estimated the prevalence of reproductive desire to be 49%, where the outcome was correlated with age, absence of children, immigration status, and not receiving ART. In Ethiopia, Melaku et al. [19] found the prevalence of reproductive desire to be 45.5%, which was associated with age, number of children, and knowledge of the woman’s serological status by her sexual partner or husband.

In Brazil, a few studies have been conducted on the prevalence and factors associated with reproductive desire in WLWHA. In one of the first Brazilian studies, a prevalence of 21% of reproductive desire among WLWHA was found in the city of São Paulo (Southeast region) [20]. In the Northeast, a study reported a prevalence of 40.0% [21]. This study also found an association between age < 30 years, reproductive desire by the partner, number of children, and knowledge of the woman’s serological status by her partner [21]. In Central Brazil, no research has been conducted on the reproductive desire of WLWHA. The present study reports important data on the determinants of reproductive desire in WLWHA, which may contribute to the implementation or strengthening of public policies focusing on the improvement of family planning and assisted reproduction services, and reproductive health care of this population. The purpose of this study was to estimate the prevalence and examine the factors associated with reproductive desire among WLWHA in Central Brazil.

## Material and methods

### Design, location, and sampling

A cross-sectional study was conducted between September 2015 and August 2016. The sample cohort consisted of WLWHA who used treatment referral services and those of non-governmental organizations (NGOs) that assist PLWHA in Goiás State, located in the Central–West region of Brazil. Inclusion criteria were: (i) women of childbearing age between 18 and 49 years [18] and (ii) a medical diagnosis of HIV/AIDS. Exclusion criteria included women who had undergone menopause despite being within the age range of this study.

Sample selection was conducted via a non-probabilistic sampling technique. For the sample calculation of this population, we considered a statistical power of 80.0% (β = 20.0%), a level of significance of 95% (α = 0.05), a drawing effect of 1.0, and a prevalence of reproductive desire of 21% in WLWHA [20]. Thus, the minimum sample size required was 274 women.

### Data collection

Participants were recruited from the outpatient clinic of referral services while awaiting clinical consultation or NGO meetings. Participants were approached for inclusion in the study in consecutively, until the desired sample size was reached. After approaching potential study participants, the women who consented to participate were interviewed face-to-face by trained health professionals. The questionnaire included items addressing sociodemographic characteristics, substance use, sexual behavior, clinical aspects, gynecological-obstetrics, reproductive desire and other potential associated factors (QNR 1, QNR 2). The questionnaire consisted of questions used in previous studies and was tested on 2% of the sample population during a pilot test.

### Variables

#### Dependent variable

The dependent variable of this study was reproductive desire expressed by the participants during the interview.

#### Independent variables

Independent variables included: age (years), categorized as < 30 years, 30–39 years, and > 40 years [22]; education (years), categorized as < 4 years, 5–8 years, and > 8 years; marital status (single, divorced/widowed or married) [18]; self-reported skin color/race (white, black, brown, or other [Native Brazilians or Asian]), categorized according to the classification of this variable for the Brazilian population by the Brazilian Institute of Geography and Statistics [23]; formal employment (yes or no) [13]; history of illicit drug use (yes or no); tobacco use in the previous month (yes or no); alcohol consumption in the previous month (yes or no) [24]; current sexual partnership (yes or no) [10]; use of condom (never, sometimes, or always) in the previous six months [5]; time since HIV diagnosis (years), categorized as < 2 years, 3–5 years, or > 5 years; current ART (yes or no) [18]; undetectable viral load (yes or no), defined as < 50 copies/mL on the last exam [25]; recent CD4 count, categorized as < 200, 200–350 or > 350 cells/mL [26]; opportunistic diseases in the previous six months (yes or no); sexually transmitted infections (STIs) in the previous six months (yes or no); number of children (none, 1–2, or > 3) [18]; previous abortion (yes or no) [26]; pregnancy after HIV diagnosis (yes or no) [22]; history of a child with positive serology (yes or no) [26]; reproductive desire of partner (yes, no, or do not know) [22]; HIV status of partner (positive, negative or do not know) [10]; awareness of assisted reproduction techniques (yes or no). All variables were based on self-reported questionnaires, except for the last viral load and CD4 counts that were obtained from the participant’s medical records.

### Statistical analysis

The data were analyzed using Stata, version 14.0 software (StataCorp LLC, College Station, TX, USA). Initially, the qualitative variables were verified by the Lilliefors-corrected Kolmogorov–Smirnov test. Descriptive analysis of variables was also performed. Quantitative variables were presented as mean and standard deviation (SD) and qualitative variables as absolute and relative frequencies. Prevalence of reproductive desire was estimated using a confidence interval of 95.0% (95.0% CI). Bivariate Poisson regression analysis was conducted to verify the factors associated with the outcome of interest. Variables with p < 0.20 were included in the Poisson regression model with robust variance to estimate adjusted prevalence ratio (APR) and respective 95% CI [27]. Values of p < 0.05 were considered as statistically significant.

### Ethical aspects

This study was approved by the Research Ethics Committee of the Federal University of Goiás (protocol number: 763.839/2014). Informed consent written was obtained from all participants prior enrollment into the study.

## Results

310 women were invited to participate in the study; 36 were excluded because they were 50 years of age or older. None of the women in the age range of the study had menopausal status. Thus, a total of 274 WLWHA participated in the study. The mean age of participants was 38.3 years (SD ± 7.72), with half (50.0%) the women aged 40 years or over. The mean education was 8.18 years (SD ± 4.35), with 46.4% of participants having more than 8 years of formal education. Approximately 45.3% were married, 50.0% self-declared brown skin color, and 54.3% had formal employment Table 1).

**Table 1:** Sociodemographic characteristics of WLWHA in Central Brazil, 2015–2016.

Variables	N = 274	%
**Age (years)**, mean ± SD^a^	38.30 ± 7.72	
< 30	44	16.1
30–39	93	33.9
≥ 40	137	50.0
**Education (years)**, mean ± SD^a^	8.18 ± 4.35	
≤ 4	61	22.3
5–8	86	31.4
> 8	127	46.4
**Marital status**		
Single	92	33.6
Divorced or widowed	58	21.2
Married	124	45.3
**Skin color/race** (self-declared)		
White	59	21.5
Black	59	21.5
Brown	137	50.0
Other^b^	19	6.9
**Formal employment**		
No	124	45.3
Yes	150	54.7

^a ^Standard deviation;

^b ^Includes Native Brazilian and Asian race.

The use of alcohol, tobacco, and illicit drugs were reported by 32.5%, 19.7%, and 12.8% of participants, respectively. The average age at first sexual intercourse was 15.86 years (SD ± 2.63). Of the total, 205 (74.8%) women reported a current sexual partnership and 42.0% reported inconsistent condom use (never or sometimes) in the previous six months. (Table 2).

**Table 2:** Substance use, sexual behaviors, and clinical characteristics of WLWHA in Central Brazil, 2015–2016.

Variables	N = 274	%
**SUBSTANCE USE**		
Alcohol^a^	89	32.5
Tobacco^a^	54	19.7
Illicit drugs^b^	35	12.8
**SEXUAL BEHAVIORS**		
Age at first sexual intercourse (years)	15.86 ± 2.63	
**Current sexual partnership**		
No	69	25.2
Yes	205	74.8
**HIV status of partner** (n = 205)		
Positive	68	33.2
Negative	110	53.7
Do not know	27	13.2
**Condom use**^d^		
Never	52	19.0
Sometimes	63	23.0
Always	159	58.0
**CLINICAL ASPECTS**		
**Route of HIV transmission**		
Sexual relationship	218	79.6
Blood transfusion	5	1.8
Vertical transmission	4	1.5
Injection drug use	1	0.4
Other	9	3.3
Do not know	37	13.5
**Time since diagnosis** (years)		
< 2	72	26.3
3–5	72	26.3
> 5	130	47.3
**Current ART**^e^		
No	32	11.7
Yes	242	88.3
**Undetectable viral load**^f^		
No	117	54.2
Yes	99	45.8
**Recent CD4 count** (cells/mL), mean ± SD^a^^,^^c^^,^^g^	612.05 ± 352.70	
≤ 200	26	12.6
200–350	27	13.0
> 350	154	74.4
**Opportunistic diseases**^h^		
Yes	47	17.2
No	227	82.8
**STIs**^i^^,^^d^^,^^j^		
Yes	44	16.3
No	226	83.7

^a ^Previous month;

^b^ Lifetime;

^c^ Standard deviation;

^d^ Previous six months;

^e^ Antiretroviral therapy;

^f^ Missing: 58;

^g ^Missing: 67;

^h^ Previous six months;

^i^ Sexually transmitted infections;

^j^ Missing: 4.

Forty-seven percent of women had been diagnosed with HIV for more than 5 years, with the main route of transmission reported as sexual transmission (79.6%). A total of 242 participants (88.3%) reported being on ART. The most recent average CD4 count was 612.05 cells/mL (SD ± 352.70), and most women (74.4%) had a CD4 cell count > 350 cells/mL. Undetectable viral load was found in 45.8% of women. Opportunistic diseases and STIs in the last 6 months were reported by 17.2% and 16.3% of women, respectively (Table 2).

Table 3 shows the reproductive variables of study participants. The mean number of pregnancies was 2.74 (SD ± 1.69), and 148 (54.0%) participants reported a history of three or more pregnancies. The mean number of children was 2.46 (SD ± 1.57) and 248 (90.5%) women reported having at least one living child. Previous abortion was reported by 17.5% of women. More than half of participants (67.1%) became pregnant after HIV diagnosis and only 3.9% reported having children with HIV-positive serology. Of the total number of women with sexual partners in the last 6 months, 38.5% reported a reproductive desire by their partners.

**Table 3:** Reproductive variables of WLWHA in Central Brazil, 2015–2016.

Variables	N = 274	%
**Total pregnancies,** mean ± SD^a^	2.74 ± 1.69	
None	19	6.9
1–2	107	39.1
≥ 3	148	54.0
**Number of children,** mean ± SD^a^	2.46 ± 1.57	
None	26	9.5
1–2	120	43.8
≥ 3	128	46.7
**Previous abortion (n = 255)**^**c**^		
No	185	72.5
Yes	70	17.5
**Pregnancy after diagnosis (n = 255)** ^**c**^		
Yes	84	32.9
No	171	67.1
**History of children with HIV-positive serology (n = 255)**^**b**^^**,**^ ^**c**^		
Yes	10	3.9
No	244	96.1
**Reproductive desire by partner** (n = 205)		
No	100	48.8
Do not know	26	12.7
Yes	79	38.5

^a^ Standard deviation;

b. Missing: 1;

c.Valid only for women who have reported a history of pregnancy.

The prevalence of reproductive desire in WLWHA was 25.9% (95.0% CI: 21.1% to 31.4%). In bivariate analysis, the outcome was statistically associated with age between 30 and 39 years (PR: 1.84; p = 0.042) and < 30 years (PR: 4.04; p < 0.001), black skin color (PR: 2.55; p = 0.017), partner's reproductive desire (PR: 9.91; p < 0.001) and, having one to two children (PR: 1.85; p = 0.032) or no children (PR: 4.92; p < 0.001) (Table 4).

**Table 4:** Potential factors associated with reproductive desire in WLWHA in Central Brazil, 2015–2016.

Variables	Reproductive desire		Crude PR^b^ (95.0% CI)^c^	p
	n/Total^a^	%		
**Age** (years)				
≥ 40	20/137	14.6	1.00	
30–39	25/93	26.9	1.84 (1.03–3.31)	**0.042**
< 30	26/44	59.1	4.04 (2.25–7.25)	**< 0.001**
**Education** (years)				
≤ 4	12/61	19.7	1.00	
5–8	19/86	22.1	1.12 (0.54–2.31)	0.753
> 8	40/127	31.5	1.60 (0.83–3.05)	0.153
**Marital status**				
Single	26/92	28.3	1.00	
Separated or widowed	9/58	15.5	0.54 (0.25–1.17)	0.121
Married	36/124	29.0	1.02 (0.62–1.70)	0.917
**Formal employment**				
No	33/124	26.6	1.00	
Yes	38/150	25.3	0.95 (0.59–1.51)	0.836
**Skin color/race** (self-declared)				
White	9/59	15.3	1.00	
Black	23/59	39.0	2.55 (1.18–5.52)	**0.017**
Brown	34/137	24.8	1.62 (0.78–3.39)	0.194
Others	5/19	26.3	1.72 (0.57–5.14)	0.328
**Current sexual partnership**				
No	11/69	15.9	1.00	
Yes	60/205	29.5	1.83 (0.96–3.49)	0.064
**Condom use**				
Never	12/52	23.1	1.00	
Sometimes	17/63	27.0	1.16 (0.55–2.44)	0.678
Ever	42/159	26.4	1.14 (0.60–2.17)	0.680
**HIV status of partner**				
Positive	17/68	25.0	1.00	
Do not know	7/27	25.9	1.03 (0.43–2.50)	0.935
Negative	36/110	32.7	1.30 (0.73–2.23)	0.360
**Tobacco use**				
Yes	17/54	31.5	1.00	
No	54/220	24.5	0.77 (0.45–1.34)	0.371
**Alcohol use**				
Yes	28/89	31.5	1.00	
No	43/185	23.2	0.73 (0.45–1.18)	0.213
**Illicit drug use**				
Yes	14/35	40.0	1.00	
No	57/239	23.8	0.59 (0.33–1.06)	0.083
**Reproductive desire by partner**				
No	6/100	6.0	1.00	
Do not know	7/26	26.9	4.48 (1.50–13.3)	**0.007**
Yes	47/79	59.5	9.91 (4.23–23.20)	**< 0.001**
**Number of children**				
≥ 3	19/128	14.8	1.00	
1–2	33/120	27.5	1.85 (1.05–3.25)	**0.032**
None	19/26	73.1	4.92 (2.60–9.29)	**< 0.001**
**History of children with HIV-positive serology**				
Yes	2/10	20.0	1.00	
No	55/244	22.5	1.12 (0.27–4.62)	0.868
**Pregnancy after diagnosis**				
Yes	18/84	21.4	1.00	
No	40/171	23.4	1.09 (0.62–1.90)	0.757
**Previous abortion**				
No	36/185	19.5	1.00	
Yes	22/70	31.4	1.61 (0.95–2.74)	0.076
**Opportunistic diseases**				
Yes	15/47	31.9	1.00	
No	56/227	24.7	0.77 (0.43–1.36)	0.376
**STIs**				
Yes	13/44	29.5	1.00	
No	57/226	25.2	0.85 (0.46–1.55)	0.607
**Time since diagnosis** (years)				
< 2	20/72	27.8	1.00	
3–5	18/72	26.4	0.95 (0.50–1.77)	0.873
> 5	32/130	24.6	0.86 (0.50–1.54)	0.672
**Current ART**				
No	8/32	25.0	1.00	
Yes	63/242	26.0	1.04 (0.49–2.17)	0.914
**Recent CD4 count**				
≤ 200	6/26	23.1	1.00	
200–350	7/27	25.9	1.12 (0.37–3.34)	0.834
> 350	42/154	27.3	1.18 (0.50–2.78)	0.702
**Undetectable viral load**				
No	26/117	22.2	1.00	
Yes	32/99	32.3	1.45 (0.86–2.44)	0.156
**Knows techniques of assisted reproduction**				
No	38/176	21.6	1.00	
Yes	33/98	33.7	1.55 (0.97–2.48)	0.062

^a^ Number of valid responses;

^b^ Prevalence ratio;

^c^ Confidence interval of 95%.

Table 5 presents the factors independently associated with reproductive desire in WLWHA. We verified that reproductive desire was associated with age < 30 years (APR: 2.93, p < 0.001), black skin color/race (APR: 2.28, p = 0.017), reproductive desire by the partner (APR: 7.55; p < 0.001), absence of children (APR: 2.13, p = 0.003), previous abortion (APR: 1.65, p = 0.045) and undetectable viral load (APR: 1.92, p = 0.043).

**Table 5:** Factors associated with reproductive desire in WLWHA in Central Brazil, 2015–2016.

Variables	APR^a^ (95.0% CI)^b^	p
**Age** (years)		
≥ 40	1.00	
30–39	1.73 (0.99–3.70)	0.106
< 30	2.90 (1.63–5.17)	< 0.001
**Skin color/race** (self-declared)		
White	1.00	
Black	2.28 (1.16–4.49)	0.017
Brown	1.65 (0.85–3.20)	0.138
Other	1.50 (0.62–3.60)	0.357
**Reproductive desire by partner**		
No	1.00	
Do not know	3.53 (1.42–8.79)	0.007
Yes	7.55 (3.47–16.42)	< 0.001
**Number of children**		
≥ 3	1.00	
1–2	1.42 (0.90–2.22)	0.123
None	2.13 (1.30–3.50)	0.003
**Previous abortion**		
No	1.00	
Yes	1.65 (1.01–2.72)	0.045
**Undetectable viral load**		
No	1.00	
Yes	1.92 (1.02–3.63)	0.043

^a^ Adjusted prevalence ratio;

^b^Confidence interval of 95%;

Note: McFadden’s R^2^: 0.246; Pearson Goodness-of-fit: p = 0.987.

## Discussion

This is the first study to evaluate the prevalence and associated factors of reproductive desire among WLWHA in Central Brazil. The results show a relatively high prevalence of reproductive desire in the sample of women in our study (25.9%). The factors associated with reproductive desire were young age, black skin color, partner's reproductive desire, no children, history of abortion, and undetectable viral load.

Previous investigations conducted in Brazil have shown a wide variation in the prevalence of reproductive desire among WLWHA. In the São Paulo State (Southeast region), one study estimated a frequency of 15.0% among 1,068 participants [28]. In Rio de Janeiro (also in the Southeast region), another study estimated a frequency of 36.0% in 181 participants [29]. In Fortaleza (Northeast region), a study found a prevalence of 40.0% for 229 women [21]. During the last decade in Brazil, factors such as improvement in the prevention of vertical transmission as well as increased survival of PLWHA have influenced the increase in reproductive desire among WLWHA [24].

Studies conducted in other countries have also found varying prevalence of reproductive desire among WLWHA. On the African continent, studies have shown varying prevalence, from 24.4% in Ethiopia to 78% in Gabon [30, 31, 32, 18, 33]. In Asia, one study found a prevalence of 34.0% in Iran [34]. In North America, prevalence ranging from 25.8% in Canada to 44.0% in the United States has been estimated [16, 34, 35]. These studies have reported differing results, which may be associated to differences in geographical location, cultural factors, prevalence of determinants, and methodological differences applied in each investigation [16, 36].

In the present study, we observed an association between young age (< 30 years) and reproductive desire, as evidenced in several other works [16, 18, 25, 26, 32]. A global meta-analysis conducted by Berhan & Berhan [37] found that age < 30 years is a strong and independent predictor of reproductive desire among PLWHA. Young age influences the reproductive desire of infected as well as uninfected women [24]. This fact is related to strong reproductive desire among most young people of reproductive age, independent of their serological status [37]. Furthermore, reproductive desire is natural during this period of life, this being the most common phase for creating the family nucleus [19, 26].

In this study, black skin color was associated with reproductive desire, different to the findings of other studies among PLWHA in Brazil, that shown absence this variable as a predictor of [20, 28, 29, 21, 38]. On the other hand, some studies conducted in other countries have shown that ethnic differences directly interfere with the reproductive desire of WLWHA [16, 17, 26]. The association of reproductive desire with black skin color lacks clear explanation because there is little research analyzing the decision in reproductive desire between Brazilian WLWHA by race. Other studies are needed to verify those mechanisms that contribute to ethnic differences in the reproductive desire of WLWHA.

The reproductive desire of participants’ partners was associated with the outcome investigated, as found in other studies [13, 25, 35]. Finocchario-Kessler et al. [29] emphasized that in relationships, children are highly valued for the emotional fulfillment they provide to the parents and for the connection generated between the couple. Children are often considered a prerequisite for a full and happy life. [39].

As observed in other studies [18, 19, 32], the absence of children was a strong determinant for reproductive desire. In addition, there was a higher prevalence of reproductive desire among women with a history of abortion. The family nucleus is important for women in Brazil, where cultural issues are directly linked to the value of motherhood [29]. Guidelines for family planning among couples living with HIV/AIDS have been described in the country [40], but with low applicability. Health professionals need to work together to develop guidelines for high quality reproductive counseling of WLWHA who intend to become pregnant [29].

Undetectable viral load was associated with reproductive desire in our sample. This result reflects a positive evaluation of the state of health, physical well-being, and capacity to cope with the stress of pregnancy among WLWHA [41], which may contribute to increased reproductive desire. These factors lead to greater safety for conception by reducing the risk of vertical HIV transmission. A study conducted in Canada [16] did not find this predictor to be significant for reproductive desire. On the other hand, studies have shown an association between CD4 cell count and reproductive desire [10, 26], which may serve as a proxy comparison for our findings.

The results of this study should be interpreted within the context of its limitations. The cross-sectional nature of this research did not allow for establishment of cause and effect relationships between reproductive desire and the predictive variables [32]. The data were self-reported, and therefore, was subject to memory and response bias. The non-probabilistic and limited sample of only women linked to health services and NGOs limits generalization of the results. Another potential limitation was that participants were not queried about their reasons for choosing not to have children. In addition, the issue of reproductive desire and fertility is a sensitive issue, which may have resulted in underestimation of the prevalence. Finally, reproductive desire is very dependent on the cultural, social, and economic factors experienced by different populations [13].

This study showed a relatively high prevalence of reproductive desire among WLWHA. These findings can contribute to the implementation and improvement of health services aimed at the sexual and reproductive health of people living with HIV/AIDS, with a greater focus on promotion and education, with the aim to promote decision autonomy on fertility and contraception issues. Continued discussion of women's reproductive choices in the context of HIV/AIDS is necessary because WLWHA have a desire to conceive in the same way as the general population. Thus, WLWHA should have the right to make decisions about conception, with support from their nuclear family and social circle, as well as access to healthcare services that concentrate on assisted reproduction, pregnancy, childbirth, and the postpartum period.

## Supporting information

S1 File Questionnare 1: English. QNR 1.
(PDF)

S2 File Questionário 2: Português. QNR 2.
(PDF)
