# Supplementary material for: Reproductive desire among women living with HIV/AIDS in Central Brazil: Prevalence and associated factors
Source: PLoS One. 2017 Oct 20;12(10):e0186267. doi: 10.1371/journal.pone.0186267 (PMC5650151; doi:10.1371/journal.pone.0186267)
Supplement: S1 File — QNR 1. (PDF) [file pone.0186267.s001.pdf]

Questionnaire 1: English

Data collection questionnaire of Study: *"Prevalence and factors associated with reproductive desire in women living with HIV/AIDS"*

**Identification number**: |__|__|__|	**Survey Date (MM/DD/YYYY) |__|__|/|__|__|/|__|__|__|__|**	
Location survey: ___________________________		
**A. Sociodemographic characteristics**		
**A1**	Name: ____	**Phone number:|__|__| |__|__|__|__|__|__|__|__|__|**
**A2**	Date of birth (MM/DD/YYYY) |__|__|/|__|__|/|__|__|__|__| Age (years): __________	[0] < 30 years<br>[1] 30-39 years<br>[2] ≥ 40 years
**A3**	Education (years): ___________	[0] < 4 years<br>[1] 5-8 years<br>[2] > 8 years
**A4**	Marital status	[0] Single<br>[1] Married<br>[2] Divorced or widowed
**A5**	Race/skin color	[0] White<br>[1] Black<br>[2] Brown<br>[3] Native Brazilian<br>[4] Asian
**A6**	Are you currently formally employed?	[0] No<br>[1] Yes
**B. Substance use**		
**B1**	Have you consumed alcohol at least once in the previous 30 days?	[0] No<br>[1] Yes
**B2**	Have you used tobacco at least once in the previous 30 days?	[0] No<br>[1] Yes
**B3**	Have you used any illicit drugs at least once in your lifetime<br>(e.g. marijuana, cocaine, crack cocaine, heroin, etc.)?	[0] No<br>[1] Yes
**C. Sexual behaviors**		
**C1**	At what age did you experience your first sexual intercourse?	Age (years): __________
**C2**	Do you currently have a sexual partner?	[0] No<br>[1] Yes
**C3**	If you answered yes to C2.<br>What is the HIV status of your current sexual partner?	[0] Positive<br>[1] Negative<br>[2] Do not know

**C4**	In the last 12 months, how often did you use a condom with your sexual partner(s)?	[0] Never<br>[1] Sometimes<br>[2] Always
**D. Clinical aspects**		
**D4**	How do you think you contracted HIV?	[0] Sexual relationship<br>[1] Blood transfusion<br>[2] Vertical transmission (mother-child)<br>[3] Injection drug use<br>[4] Other means<br>[5] Do not know
**D5**	How long have you been diagnosed by a doctor as HIV positive?	[0] < 2 years<br>[1] 3-5 years<br>[5] > 5 years
**D6**	Are you currently being treated with antiretroviral therapy?	[0] No<br>[1] Yes
**D7**	In the previous six months, have you been diagnosed with an opportunistic illness (except sexually transmitted infections) by a doctor?	[0] No<br>[1] Yes
**D8**	In the previous six months, have you been diagnosed with any sexually transmitted infections by a doctor?	[0] No<br>[1] Yes
**D9**	*Medical record consultation*: Result of the last CD4 cell count: _______ cells/mL	[0] < 200 (cells/mL)<br>[1] 200-350 (cells/mL)<br>[2] > 350 (cells/mL)
**D10**	*Medical record consultation*: Undetectable viral load in the last exam (defined as < 50 copies/mL)	[0] No<br>[1] Yes
**E. Gynecological-obstetrics data**		
**E1**	Total number of pregnancies: ___________<br>Please answer questions E3-E5 if you have had one or more pregnancies.	[0] None<br>[1] 1-2<br>[2] ≥ 3
**E2**	Number of live children	[0] None<br>[1] 1-2<br>[2] ≥ 3
**E3**	Have you ever had an abortion?	[0] No<br>[1] Yes
**E4**	Have you become pregnant after being diagnosed with HIV diagnosis?	[0] No<br>[1] Yes
**E5**	Do you have or have you had any HIV-positive children?	[0] No<br>[1] Yes
**F. Reproductive desire and others potential associated factors**		
**F1**	Would you like to have children in the future?	[0] No<br>[1] Yes
**F2**	If you have a partner, does your partner want to have children?	[0] No<br>[1] Yes

		[2] Do not know
**F3**	Do you know about assisted reproduction techniques for people living with HIV/AIDS?	[0] No<br>[1] Yes
