# Supplementary material for: Reproductive desire among women living with HIV/AIDS in Central Brazil: Prevalence and associated factors
Source: PLoS One. 2017 Oct 20;12(10):e0186267. doi: 10.1371/journal.pone.0186267 (PMC5650151; doi:10.1371/journal.pone.0186267)
Supplement: S2 File — QNR 2. (PDF) [file pone.0186267.s002.pdf]

Questionário 2: Português

Questionário de coleta de dados do estudo “*Prevalência e fatores associados com o desejo reprodutivo em mulheres vivendo com HIV/AIDS”*.

<table>
<tr><td colspan="2">Número de identificação: |__|__|__|</td><td>Data (DIA/MÊS/ANO) |__|__|/|__|__|/|__|__|__|__|</td></tr>
<tr><td colspan="3">Local do estudo: ____________________________</td></tr>
<tr><td colspan="3">A. Características sociodemográficas</td></tr>
<tr><td>A1</td><td>Ano: ____</td><td>Número de telefone:
|__|__| |__|__|__|__|__|__|__|__|__|__|</td></tr>
<tr><td>A2</td><td>Data de Nascimento (DIA/MÊS/ANO) |__|__|/|__|__|/|__|__|__|__|
Idade (anos): __________</td><td>[0] < 30 anos
[1] 30-39 anos
[2] ≥ 40 anos</td></tr>
<tr><td>A3</td><td>Escolaridade (anos): ___________</td><td>[0] < 4 anos
[1] 5-8 anos
[2] > 8 anos</td></tr>
<tr><td>A4</td><td>Estado marital</td><td>[0] Solteira
[1] Casada
[2] Divorciada ou viúva</td></tr>
<tr><td>A5</td><td>Raça/Cord a pele</td><td>[0] Branca
[1] Preta
[2] Parda
[3] Indígena
[4] Amarela</td></tr>
<tr><td>A6</td><td>Você está, atualmente, empregada formalmente?</td><td>[0] Não
[1] Sim</td></tr>
<tr><td colspan="3">B. Uso de substâncias</td></tr>
<tr><td>B1</td><td>Você consumiu álcool pelo menos uma vez nos últimos 30 dias?</td><td>[0] Não
[1] Sim</td></tr>
<tr><td>B2</td><td>Você usou tabaco pelo menos uma vez nos últimos 30 dias?</td><td>[0] Não
[1] Sim</td></tr>
<tr><td>B3</td><td>Você usou drogas ilícitas pelo menos uma vez na sua vida
(por exemplo: maconha, cocaína, crack, heroína, etc.)?</td><td>[0] Não
[1] Sim</td></tr>
<tr><td colspan="3">C. Comportamentos sexuais</td></tr>
<tr><td>C1</td><td>Em que idade você experimentou sua primeira relação sexual?</td><td>Idade (anos): __________</td></tr>
<tr><td>C2</td><td>Você tem atualmente um parceiro sexual?</td><td>[0] Não
[1] Sim</td></tr>
<tr><td>C3</td><td>Se você respondeu sim a C2 question.
Qual é o status de HIV do seu parceiro sexual atual?</td><td>[0] Positivo
[1] Negativo
[2] Não sei</td></tr>
</table>

**C4**	Nos últimos 12 meses, com que frequência você usou um preservativo com seu(s) parceiro(s) sexual (s)?	[0] Nunca<br>[1] As vezes<br>[2] Sempre
**D. Aspecos clínicos**		
**D4**	Como você acha que contraiu o HIV?	[0] Relação sexual<br>[1] Transfusão sanguínea<br>[2] Transmissão vertical (mãe para filho)<br>[3] Uso de droga injetável<br>[4] Outros meios<br>[5] Não sabe
**D5**	Há quanto tempo você foi diagnosticado por um médico como HIV positivo?	[0] < 2 anos<br>[1] 3-5 anos<br>[5] > 5 anos
**D6**	Você está sendo tratada atualmente com terapia antirretroviral?	[0] Não<br>[1] Sim
**D7**	Nos seis meses anteriores, você foi diagnosticado com uma doença oportunista (exceto infecções sexualmente transmissíveis) por um médico?	[0] Não<br>[1] Sim
**D8**	Nos seis meses anteriores, você foi diagnosticado com alguma infecção sexualmente transmissível por um médico?	[0] Não<br>[1] Sim
**D9**	*Consulta de prontuário médico:* Resultado da última contagem de células CD4*:*_______ células/mL	[0] < 200 (células/mL)<br>[1] 200-350 (células/mL)<br>[2] > 350 (células/mL)
**D10**	*Consulta de prontuário médico*: Carga viral indetectável no último exame (definido como < 50 cópias/mL)	[0] Não<br>[1] Sim
**E. Dados ginecológicos e obstétricos**		
**E1**	Número total de gravidezes: ___________<br>Por favor, responda as perguntas E3-E5 se tiver tido uma ou mais gravidezes.	[0] Nenhuma<br>[1] 1-2<br>[2] ≥ 3
**E2**	Número de filhos vivos: ______________	[0] Nenhuma<br>[1] 1-2<br>[2] ≥ 3
**E3**	Você já teve um aborto?	[0] Não<br>[1] Sim
**E4**	Você ficou grávida depois de ter sido diagnosticada com diagnóstico de HIV?	[0] Não<br>[1] Sim
**E5**	Você teve ou você teve filhos com HIV?	[0] Não<br>[1] Sim
**F. Desejo reprodutivo e outros potenciais fatores associados**		
**F1**	Você gostaria de ter filhos no futuro?	[0] Não<br>[1] Sim
**F2**	Se você tem um parceiro, seu parceiro gostaria de ter filhos?	[0] Não<br>[1] Sim

		[2] Não sei
**F3**	Você conhece técnicas de reprodução assistida para pessoas que vivem com HIV/AIDS?	[0] Não<br>[1] Sim
